# Community-based dementia risk management and prevention program for Aboriginal Australians (DAMPAA): a randomised controlled trial study protocol

**DOI:** 10.1136/bmjopen-2024-088281

**Published:** 2024-09-13

**Authors:** Alex Lalovic, Glennette Dowden, Lesley Markey, Michael Bynder, Lynette Yappo, Kay L Cox, Irene Mateo-Arriero, Leon Flicker, Dawn Bessarab, Sandra Thompson, Carmel Kickett, Deborah Woods, Carmela F Pestell, Paula Edgill, Keith Hill, Christopher Etherton-Beer, Dina LoGiudice, Osvaldo P Almeida, Ivan Lin, Rachel Milte, Julie Ratcliffe, Zoë Hyde, Kate Smith

**Affiliations:** 1 Centre for Aboriginal Medical and Dental Health, The University of Western Australia, Perth, Western Australia, Australia; 2 Western Australian Centre for Health and Ageing, The University of Western Australia, Perth, Western Australia, Australia; 3 Western Australian Centre for Rural Health, The University of Western Australia, Geraldton, Western Australia, Australia; 4 Moorditj Koort Aboriginal Corporation, Perth, Western Australia, Australia; 5 Geraldton Regional Aboriginal Medical Service, Geraldton, Western Australia, Australia; 6 School of Psychological Science, University of Western Australia, Perth, Western Australia, Australia; 7 Derbarl Yerrigan Health Service Aboriginal Corporation, Perth, Western Australia, Australia; 8 Rehabilitation Ageing and Independent Living Research Centre, Monash University, Melbourne, Victoria, Australia; 9 Department of Medicine - Royal Melbourne Hospital, The University of Melbourne, Melbourne, Victoria, Australia; 10 Institute for Health Research, The University of Notre Dame Australia, Perth, Western Australia, Australia; 11 Health and Social Care Economics Group, Caring Futures Institute, College of Nursing and Health Sciences, Flinders University, Adelaide, South Australia, Australia

**Keywords:** Cognition, Dementia, Quality of Life

## Abstract

**Introduction:**

Aboriginal and Torres Strait Islander peoples are the First Peoples of Australia. Up to 45% of dementia in these populations is due to potentially modifiable risk factors. The Dementia Prevention and Risk Management Program for Aboriginal Australians (DAMPAA) is an Aboriginal Health Practitioner led programme that aims to reduce cognitive decline and functional impairment in older Aboriginal people.

**Methods:**

Design: DAMPAA is a multisite, randomised controlled trial aiming to deliver and evaluate a culturally appropriate risk factor management programme. Population: Community-dwelling Aboriginal people aged 45–90 years. Intervention: Participants will be randomly assigned to either usual care (control) or to a group programme comprising exercise and health education yarning sessions and pharmacist-delivered medication reviews delivered over a 12-month period. Primary outcome: Cognitive function (Kimberley Indigenous Cognitive Assessment (KICA)-Cog score), daily function (KICA-Activities of Daily Living (ADL) score) and quality of life (Good Spirit, Good Life and EQ-5D-5L scores). Secondary outcomes*:* Process evaluation interviews, cardiovascular risk factors, falls and death. Process evaluation will be conducted with qualitative methods. Quantitative outcomes will be analysed with generalised linear mixed models.

**Ethics and dissemination:**

The study was approved by the Western Australian Aboriginal Health Ethics Committee and the University of Western Australia Human Research Ethics Committee. Study results will be published in peer-reviewed journals and presented at scientific meetings. We will also develop and disseminate a comprehensive DAMPAA toolkit for health services. The study’s findings will guide future prevention strategies and outline a comprehensive process evaluation that may be useful in other Aboriginal health research to contextualise findings.

**Strengths and limitations of this study**

- Theory of change framework for co-design and process evaluation in partnership with Aboriginal Community Controlled Health Organisations.
- Aboriginal research methodologies including co-design workshops with Aboriginal Elders and community-controlled organisations, Aboriginal health practitioner-led programme, yarning education methods and Elders Governance Group.
- Randomised controlled trial design limits the flexibility required in Aboriginal health research.
- Lack of culturally specific neuropsychological test norms.
- Potential for experimental contamination due to the extended family structure of Aboriginal people.

## Introduction

### Background and rationale

 Aboriginal and Torres Strait Islander peoples (hereafter respectfully referred to as Aboriginal people^1^) are the First Peoples of Australia and the spiritual and cultural custodians of the land. Aboriginal Elders play a vital role in preserving traditional knowledge, language and culture.^2^ Their cognitive health is vital to enable them to preserve and teach culture, language and law to younger generations. As one Wunambal man said, ‘My grandfather, he still got all that memory in his head, and now he’s sharing that with us young fellas so that we can carry his way, the way he taught us, so that it can always be there for our kids, and their kids’.^3^

Dementia is a broad term for a group of conditions that affect cognitive functioning. Approximately 50 million people worldwide live with dementia, and this is expected to triple by 2050.^4^ Previously, identification of dementia in Aboriginal people was hampered due to the lack of a valid dementia screen. The Kimberley Indigenous Cognitive Assessment (KICA-Cog) was developed and validated in 2003 with Aboriginal people in the Kimberley region of Western Australia.^3 5^ This led to further research revealing that Aboriginal people experience dementia at a rate three to five times higher than the non-Aboriginal population—one of the highest in the world.^6^,^9^ The KICA has since been adapted and validated in other regions of Australia^8 10^ and adapted and validated for Torres Strait Islander peoples^9^ and for First Nations peoples internationally.^11 12^

While there is currently no cure for dementia, international studies have identified that up to 40% of dementia risk factors are potentially modifiable.^4^ A recent analysis identified that up to 45% of the dementia burden for Aboriginal and Torres Strait Islander peoples may be due to potentially modifiable risk factors.^13^ Potentially modifiable risk factors attributed to the high incidence of dementia in Aboriginal people include physical inactivity, high blood pressure, diabetes, head injuries, current smoking, obesity, stroke, epilepsy, polypharmacy, poor mobility and falls.^7 14 15^ In 2018–2019, 31% of Aboriginal and Torres Strait Islander adults had high blood pressure and 13% had diabetes or hyperglycaemia, while 71% of those aged ≥15 years were overweight or obese and 37% were daily smokers. Only 12% of adults living in non-remote areas met physical activity guidelines.^16^ There is a need for culturally safe health programmes targeting these modifiable risk factors.

Physical inactivity is a major risk factor for disease burden in Aboriginal people,^17^ while physical activity that starts in mid to late life can still significantly reduce the risk of cognitive decline.^18^ Regular physical activity has numerous benefits on cognition due to increased cerebral blood flow,^19^ neurogenesis and angiogenesis, synaptic plasticity, reduced inflammation and improved brain resilience.^20 21^ Physical activity interventions have demonstrated a decrease in cognitive decline^22^ and have preventive effects on cardiovascular disease, hypertension, diabetes, hypercholesterolaemia and obesity.^23^ Additionally, physical activity reduces falls risk,^24^ improves mobility^23^ and enhances quality of life.^25^ Australian health guidelines recommend 150 min of physical activity per week, including cardiovascular, resistance, mobility and balance components for older adults.^26^

Guidelines for physical activity programmes have been developed for people with mild cognitive impairment and other risk factors for dementia.^27^ Health programmes run by Aboriginal Community-Controlled Organisations (ACCOs) have been effective in improving specific health measures such as blood pressure, body mass index (BMI) and quality of life.^28 29^ Additionally, group and community activities centred on social interaction are preferred formats of physical activity for Aboriginal people,^30^ and when paired with culturally appropriate and relevant health education can lead to positive outcomes.^28 29^ Health messages delivered by Aboriginal people have a more profound impact on health behaviour^31^ and should underpin health education within Aboriginal communities. Yarning is a culturally respectful approach that Aboriginal people use to interact, convey knowledge and exchange narratives, either in a casual or formal setting, with one or multiple individuals.^32^ It is a method that can be used in Aboriginal health research and in the clinical environment to facilitate health education within Aboriginal communities.^33^ Social contact is a protective factor for dementia,^4^ with Aboriginal people emphasising the importance of strong social connections for a better quality of life in older age.^34 35^

Aboriginal health research occurs in complex contexts that present a unique number of organisational and community challenges.^36^ Staff shortages and absences, as well as high workforce turnover, particularly outside urban settings, are common challenges.^37^ Challenges for researchers include managing a project across distant locations, low recruitment numbers and providing an appropriate level of support to Aboriginal research staff to successfully carry out project objectives.^37^ Participant challenges to involvement in research include family commitments, attending funerals, caring for grandchildren, transport and other medical appointments which make it more difficult to prioritise commitments to a programme.^38^ In addition, dementia risk factor awareness in the Aboriginal community is low.^39^ Participant enablers for adherence to physical activity programmes include location and attending an Aboriginal centre with other Aboriginal people, transport assistance, enjoyment in programme activities and feeling healthy.^29 40 41^ A clear programme structure, access to varied exercise equipment and learning about health are also factors that enable increased adherence.^42^

A multi-domain, culturally safe and responsive programme specifically targeting modifiable risk factors to reduce cognitive decline in older Aboriginal people is required. Key principles for successful research with Aboriginal people include leadership and ownership by Aboriginal researchers, local development, commitment, control, supporting workers’ professional development, Aboriginal community capacity building and facilitating enablers to increase community participation.^29 37 42 43^

This paper describes the protocol for the Dementia Prevention and Risk Management Program for Aboriginal Australians (DAMPAA), co-designed to address the modifiable risk factors for dementia in Aboriginal people. The first participant was enrolled in October 2020 and the last enrolment was October 2023. Data collection will conclude in late 2024.

### Aims of the study

The overall aim of the DAMPAA project is to co-develop, deliver and evaluate a targeted culturally appropriate Aboriginal Health Practitioner (AHP) coordinated risk factor management programme to reduce cognitive decline and functional impairment in Aboriginal Australians aged 45 years and over at risk of dementia. This will be achieved by (1) co-designing a dementia risk reduction programme with and for Aboriginal people, and (2) conducting a randomised controlled trial comparing DAMPAA with usual care.

### Trial design

The DAMPAA trial is a multisite, randomised controlled trial, with randomisation at the individual level. In partnership with Elders and ACCOs in Western Australia (WA), we co-designed the AHP-led health programme for Aboriginal people aged ≥45 years using a Theory of Change (ToC) approach, as previously described.^38^

**Figure 1:**
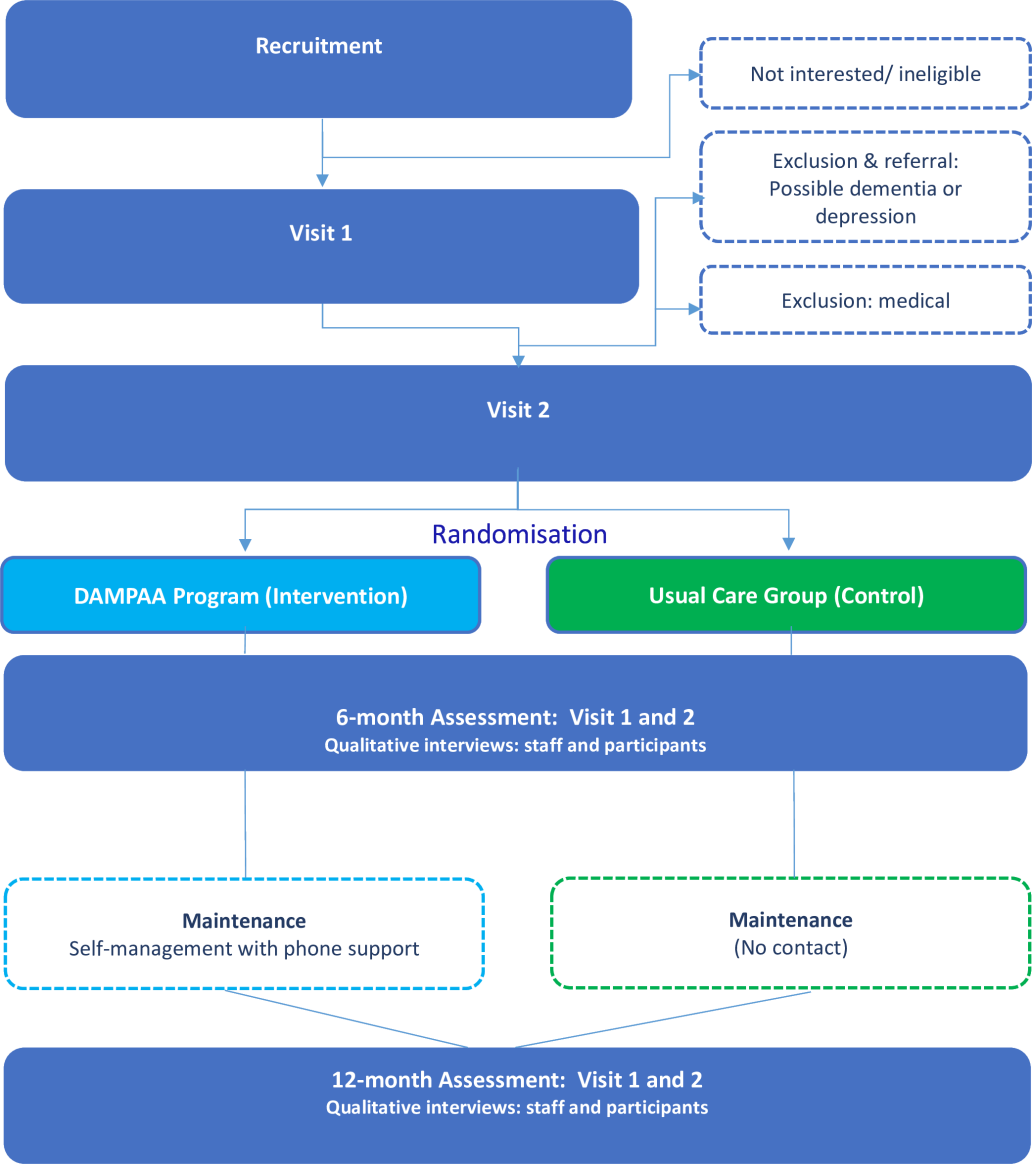
Dementia Prevention and Risk Management Program for Aboriginal Australians study participant journey.

Process evaluation of the DAMPAA programme will occur through the ToC framework, using a qualitative approach.^38^ The COVID-19 outbreak in 2020 and subsequent restrictions led to the suspension of the pilot programme and study from March 2020 to September 2021. After the recommencement of the study, various WA government health restrictions limited research activities until March 2022 when all restrictions were removed. This paper describes the protocol of the DAMPAA programme post COVID-19 suspension. Figure 1 outlines the steps involved for participants joining the programme.

## Methods

### Programme sites

Perth is the capital city of WA. Three programme sites were established across WA, with two in the Perth metropolitan area and one in the regional town of Geraldton with project partners.

### Participants

Inclusion criteria include: (1) Aboriginal person; (2) aged between 45 and 90 years; (3) community-dwelling; and (4) normal cognition (KICA-Cog score 38–39) or cognitive impairment without dementia (KICA-Cog score 31–37 without functional decline due to dementia). Exclusion criteria include: (1) dementia diagnosis; (2) presence of medical condition that restricts walking without assistance; (3) unstable or life-threatening medical condition; and (4) medical condition that contraindicates moderate physical activity, including morbid obesity (BMI≥40 kg/m^2^).

### Patient and public involvement

DAMPAA was co-designed with Elders and ACCOs. This process included elder yarning groups, workshops at three Aboriginal community controlled health services and a pilot programme run in Geraldton and Perth, WA.

### Terminology

The terms DAMPAA programme (intervention) and usual care (control) will be used in this paper based on the recommendation of our Aboriginal Elders Governance Group, who stated that ‘intervention’ and ‘control’ are not culturally safe terms in the Aboriginal community. This culturally safe terminology has also been followed in our study protocols and training manuals.

### DAMPAA group and programme

Participants randomised to the DAMPAA programme will complete the 12-month programme and assessments described later in this paper. The programme consists of two periods: the action period (1–6 months) and maintenance period (7–12 months). During the action period, participants will attend two group walking and yarning sessions per week at an ACCO and complete a third exercise-only session at home. Each session will be up to 90 min in duration. Participants will therefore be expected to engage in up to 270 min of DAMPAA programme activity per week. Sessions will be led by a trained exercise professional and AHP and will include a warm-up, walk, strength and balance exercises, stretching and cool down. The first 8 weeks of the programme will gradually acclimatise participants to exercise, with incremental increases in intensity each week. Session intensity will be monitored using the 1–10 rate of perceived exertion (RPE) measure and heart rate measures using Polar M200 heart rate watches. Participants will be set a target heart rate range each session that is individually calculated based on age and heart rate reserve. Each participant will complete a personalised goal-setting activity within the first 2 weeks and these goals will be reviewed every 4 weeks by the AHP.

DAMPAA also includes health education delivered from week 5 onwards during the physical activity session in a group yarning style. Sessions will be delivered by the AHP and exercise professional in a semi-structured yarning style method. Sessions include topics on safety and benefits of walking and yarning, diabetes prevention and management, falls prevention, good tucker (nutrition), well-being and connection and community service access. Medication reviews will be conducted by a pharmacist during the action period in a face-to-face meeting with each participant at the relevant study site. Each participant’s general practitioner (GP) will be sent a letter detailing the outcome of the review, which may include recommendations for medication changes. The study coordinator will contact GPs to determine if recommended changes were made. Participant attendance will be recorded to measure adherence to sessions, while an AHP and exercise professional will ask if the participant completed home session/s, as well as the RPE to measure intensity of the home session. Strategies to maintain adherence to the programme include providing transport; building group connection through yarning, group-themed shirts and encouraging a buddy system for home sessions; group walking outside in nature when possible, phone call reminders the day before each session and providing a culturally safe inclusive environment with Aboriginal and non-Aboriginal health professional research staff that have undergone cultural sensitivity training and are experienced working with Aboriginal people. At the conclusion of the action period, participants will complete a 6-month follow-up assessment consisting of visits 1 and 2 (figure 1). The maintenance period will consist of three walking and yarning sessions per week with all sessions completed by the participants at home or in community parks or other programmes. Participants will be provided with a file containing their weekly programme, instruction sheets and a monthly activity calendar. DAMPAA staff will monitor weekly progress through a telephone call or text message. Staff will ask participants how many walking and yarning sessions they completed that week, and the RPE for each session to measure the intensity. At the end of the maintenance period, participants will complete a 12-month assessment consisting of visit 1 and 2 and process evaluation interviews regarding their assessment and programme experiences.

### Usual care group

Participants randomised to the usual care group will be followed-up with 6 and 12-month assessments (repeat of visit 1 and visit 2 on each occasion). Participants are sent a general dementia education pamphlet via mail within 2 weeks of randomisation by the Aboriginal health practitioners. The pamphlet contains brief information on dementia risk factors for Aboriginal people.

#### Outcomes

The primary outcomes will be cognition (measured by the KICA-Cog), daily function (measured by the KICA-Function tool) and quality of life (measured by the Good Spirit, Good Life (GSGL)^34 35^ andEQ-5D-5L tools.^44^ Secondary outcomes are described in table 1. Outcome measures will be recorded at baseline, 6 months and 12 months. Death will be followed-up with next of kin.

**Table 1:** Secondary outcomes of the Dementia Prevention and Risk Management Program for Aboriginal Australians study

Secondary outcomes	Measure
Process outcomes (assumptions, resources and activities)	Yarning circles, surveys, in-depth interviews with staff members and participants
Death	Follow-up with next of kin
Cardiovascular risk factors	Resting mean systolic blood pressure (mm Hg). Mean cholesterol level (mmol/L). Glycosylated haemoglobin. Body weight, body composition and BMI (kg/m^2^). Waist and hip girths (cm). Smoking status – KICA smoking questionnaire. Alcohol intake – KICA alcohol questionnaire. Functional capacity (2 minute walk test). Regular physical activity - Rapid Assessment of Physical Activity. Medications – current medications and adherence.
Falls	Number of falls in previous 6 months

BMIbody mass indexKICAKimberley Indigenous Cognitive Assessment

**Figure 2:**
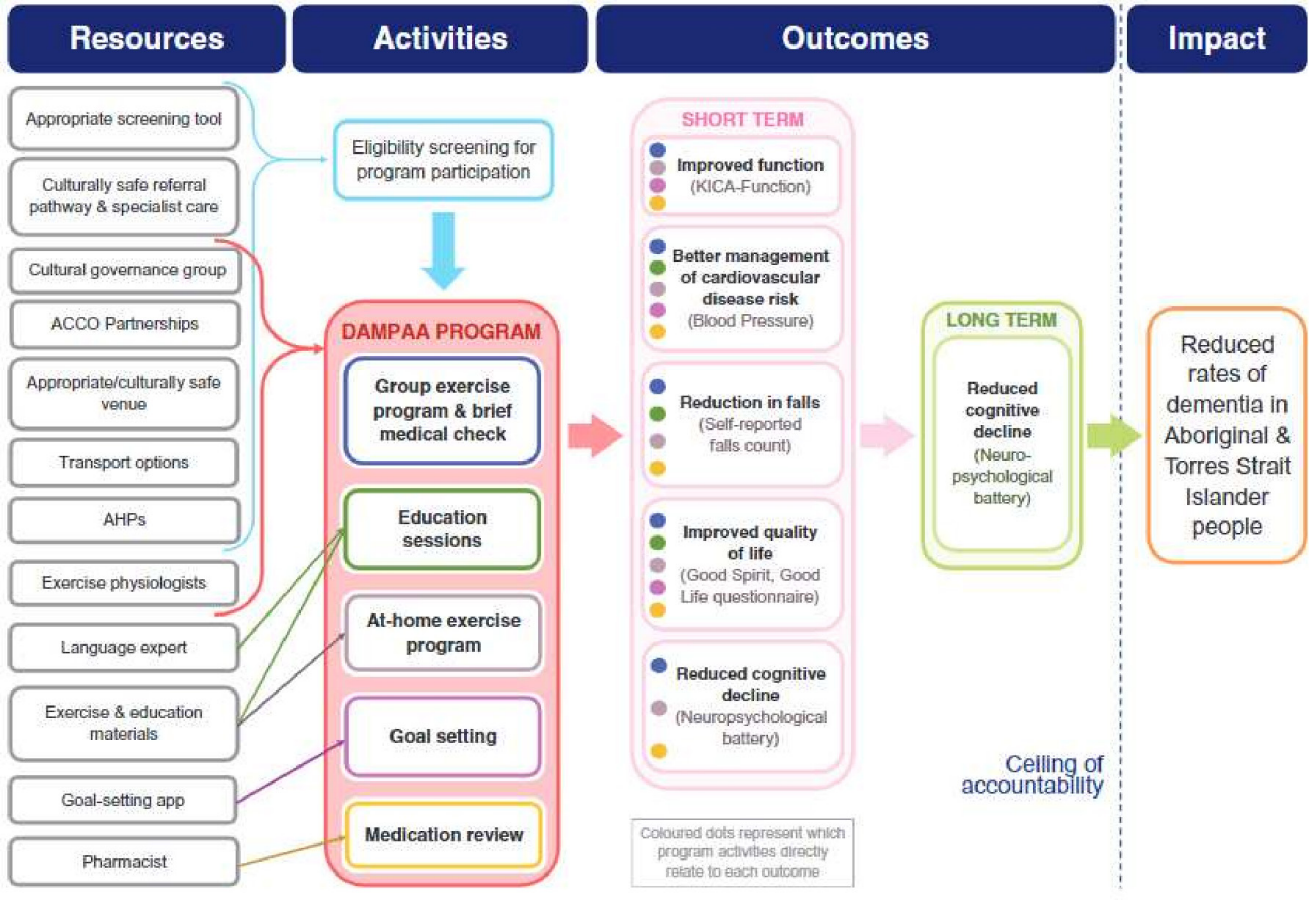
DAMPAA Theory of Change from Mateo-Arriero *et al*.^38^ ACCO, Aboriginal Community-Controlled Organisations; AHP, Aboriginal Health Practitioner; DAMPAA, Dementia Prevention and Risk Management Program for Aboriginal Australians; KICA, Kimberley Indigenous Cognitive Assessment.

Process outcomes will include the successful incorporation of (1) all DAMPAA ToC resources (figure 2) into the programme, (2) meeting of DAMPAA ToC assumptions (table 2) and (3) appropriate completion of ToC activities.^38^ These three steps will be explored through in-depth interviews with project staff and through surveys and/or yarning groups with participants.

**Table 2:** DAMPAA ToC assumptions and desired outcomes

DAMPAA ToC assumptions for resources to produce activities	DAMPAA ToC assumptions for activities to produce outcomes
Screening tool used is accepted and valid. Exercise professionals and AHPs are available for recruitment and running the programme. Exercise professionals and AHPs are appropriately trained to run the programme following the protocol.	Sufficient programme attendance from participants.Ability to come together safely for the group walking and yarning programme: Cultural safety. Health. COVID-19. Personal safety. Environment. Having the time, safe environment and space to complete the at-home exercise programme.Appropriate exercise level for participants.Appropriate content and delivery of the education, walking and yarning sessions.General practitioners enacting changes in medication based on pharmacist review.Setting achievable goals and participants taking action to reach said goals.

AHPAboriginal Health PractitionerDAMPAADementia Prevention and Risk Management Program for Aboriginal AustraliansToCTheory of Change

The DAMPAA ToC assumptions need to be met in order for the DAMPAA activities to produce the desired outcomes.^38^ These are shown in table 2.

#### Sample size

Based on unpublished analyses of a cohort study of ageing well,^7^ we expect KICA-Cog scores to decrease by a mean of 2 points in the usual care group, and by 1 point in the DAMPAA programme group, with a common SD of 3 points. A sample size of 286 people (143 in each group) would be required to provide 80% power to detect this difference, given a two-sided significance level of 5%. For the secondary outcome of daily function, we expect that 60% of the usual care group would have a decline in at least one activity of daily living during the study period, compared with only 44% of people in the DAMPAA programme arm. A sample size of 304 people would provide 80% power to detect such a difference at the 5% significance level. For the quality of life outcomes, based on previous research^35^ we expect mean GSGL and EQ-5D-5L scores to increase by 2 points and 1 point, respectively, in the DAMPAA group and to remain unchanged in the usual care group. Assuming a common SD of 3 points, a sample size of 74 and 286 people, respectively, is required to have 80% power to detect these differences at the 5% significance level. To account for potential attrition, we plan to recruit a total of 320 people (160 people per group).

#### Recruitment

Yarning workshops such as Elders yarning groups will be organised in collaboration with ACCOs to discuss the study for participant recruitment. Referral pathways with ACCOs will provide an opportunity for medical practitioners to refer interested clients to the DAMPAA team for follow-up. The DAMPAA programme will be explained in detail to participants at yarning workshops, highlighting the benefits and risks of taking part. Participants who agree to take part after meeting initial inclusion criteria will provide written informed consent to participate in the study. Participants can withdraw from the study at any time and this will be recorded with a participant withdrawal form. Forms are provided as online supplemental material 1.

#### Randomisation and blinding

Participants will be randomised into the DAMPAA programme or usual care. Randomisation was performed with Stata SE V.15.1 (StataCorp, College Station, Texas, USA) using the ralloc package^45^ and was stratified by site. We aimed for a total of 140 allocations in each of the 3 strata, with 2 treatment arms, a 1:1 treatment allocation and 3 block sizes (varying between 6, 8 and 10). Block sizes were allocated in unequal proportions. Owing to the variable block size, the randomisation process yielded 434 unique study identifiers (n=148 for site 1, n=140 for site 2 and n=146 for site 3). An independent staff member from outside the study will be responsible for drawing out each participant’s ID for allocation to a group. AHP staff will enrol participants, and allocations to DAMPAA and usual care will be drawn from sealed envelopes by an external colleague independent of the study.

Psychologist assessors are blinded to the participant group and will not assess the same participant at multiple time periods. Project staff delivering the DAMPAA programme and conducting health and fitness assessments will not be blinded. At the conclusion of the trial, we plan to offer participants in the usual care group a chance to experience the DAMPAA programme, which will continue to be delivered by ACCOs.

#### Data management and analysis

Data will be stored securely on a protected University of Western Australia (UWA) Institutional Research Data Store. Data will be de-identified to maintain confidentiality. All quantitative data will be entered and analysed using the Stata statistical package, while qualitative data will be managed with NVivo (Lumivero, Denver, Colorado). The study will employ an intention-to-treat analysis including all randomised participants. This means that participants will be analysed as per their randomisation, rather than the treatment they ultimately receive. We will use a generalised linear mixed model to compare changes in the KICA-Cog (cognition, primary outcome measure) over time between the two groups, with a significantly greater decrease in KICA-Cog scores expected in the usual care group at 12 months. The covariates in this model will be the baseline KICA-Cog score, time (6 or 12, entered as a categorical variable), group (DAMPAA programme or usual care) and an interaction between group and time. Participant and site will be treated as random effects. Similar models will be run for the other outcomes which are a mixture of variable types (proportions, counts, etc). We will handle missing data by performing complete case analyses. Results will be reported in accordance with the Consolidated Standards of Reporting Trials statement.^46^

### Harms—adverse event reporting

A formal data monitoring committee will not be established. However, any adverse events will be recorded and for serious adverse events the relevant ethics committees will be notified. No interim analyses or trial auditing is planned.

#### Baseline assessments

Baseline assessments consist of two separate assessment sessions. The first assessment, visit 1 consists of two components, initial screening and further neuropsychology assessment and will take approximately 2 hours. Assessments will be conducted in a quiet room, with support of an AHP. Assessors will have time prior to the session to meet and build rapport with participants over morning tea, and after participants complete the initial screening. The screening is made up of the KICA-Cog and function, height, weight and BMI. These tests will be administered by the AHPs and exercise professionals. Although the KICA-Cog has strong sensitivity and specificity for dementia, it is a screening measure and does not incorporate a reliable test of executive function.^5^ Consequently, to ensure cognitive abilities (ie, attention, learning/memory, executive functioning) will be further assessed, an abbreviated neuropsychological test battery was developed by the research team with neuropsychologist Pestell (CFP). The neuropsychology battery is outlined in table 3, and will be administered in that order. We selected tests that would be as culturally appropriate as possible (eg, non-verbal). The Elders Governance Group gave feedback on each test, and the tests were then trialled by AHP GD and investigator Smith on 3 pilot participants, prior to the piloting with 10 further participants. Participants who score 31 or more out of 39 on the KICA-Cog, with no functional limitations and have a BMI under 40 are eligible to continue in the study and will subsequently complete the neuropsychology assessments in the same session. These will be administered by qualified provisional psychologists undertaking postgraduate neuropsychology studies under the supervision of CFP. Participants who score 30–33 with functional limitations will be referred for review by the study geriatrician (LF) based at a new Derbarl Yerrigan Health Service memory clinic. Participants with scores outside the KICA and BMI range will be excluded and referred for further medical review where appropriate. At the completion of visit 1, eligible participants will be booked in for visit 2, which includes health questionnaires and physical fitness assessments.

**Table 3:** An overview of the visit 1 and visit 2 assessments

Visit 1 (screening)	Visit 1 (neuropsychological assessment) – if eligible	Visit 2 (health assessment)	Visit 2 (physical assessment)
Medications summary	Montreal Cognitive Assessment	Bloods draw	Body composition and girths
Blood pressure and heart rate measurement	WMS-III Spatial Span subtest	Smoking history	Grip Strength Test
Height, weight and BMI	Hopkins Verbal Learning Test	Alcohol history and current alcohol consumption	Timed Up and Go Test
Charlson Comorbidity Index	Colour Trails Test	Mini Nutritional Assessment	Sit to Stand Test (five times)
KICA-Cog	Symbol Digit Modalities Test	RAPA	Balance step test
KICA-Function	D-KEFS Design Fluency Test	Elderly Falls Screening Test (FROP Com-Screen)	5-metre walk test and 2 minute walk test
	EQ-5D-5L		
	Generalised Anxiety Disorder		
	KICA-Depression Scale		
	GSGL		

BMIbody mass indexD-KEFSDelis-Kaplan Executive Function SystemEQ-5D-5L EuroQol-5D-5LFROP-ComFalls Risk for Older People in the CommunityGSGLGood Spirit, Good LifeKICAKimberley Indigenous Cognitive AssessmentRAPARapid Assessment of Physical ActivityWMS-IIIWechsler Memory Scale (third edition)

Interviews with participants will be completed to explore Aboriginal perspectives on the DAMPAA neuropsychological assessment battery cognitive tests and testing procedures, and their acceptability.

Visit 2 testing will be administered by the AHP and an exercise professional at a venue with a quiet room and space for the physical assessments. The session will take approximately 2 hours, with a short break between the completion of the health questionnaires and start of the physical assessments. Visit 1 and 2 will be repeated at 6 and 12 months. Table 3 provides a list of all baseline assessments in visits 1 and 2.

#### Neuropsychology assessments

The MOCA consists of tasks that evaluate skills related to visuospatial/executive functions, memory, attention, language, abstraction and orientation for screening of cognitive impairment.^47^ Spatial span (forwards and backwards) will be employed from the Wechsler Memory Scale third edition to assess visual attention and spatial working memory.^48^ The Hopkins Verbal Learning Test is a brief verbal learning and memory test with recognition and recall trials.^49^ The Colour Trails Test is a non-verbal cross-cultural test that assesses sequencing, cognitive flexibility and sustained/divided attention.^50^ The Symbol Digit Modalities Test gauges attention, visual scanning, working memory and speed of processing.^51^ The Delis-Kaplan Executive Function System (DKEFS) Design Fluency is a timed task that assesses visual planning, cognitive flexibility and fluency in the generation of visual patterns.^52^ The EQ-5D-5L will be used to measure health-related quality of life.^44^ The Generalised Anxiety Disorder-7 will be used to screen for generalised anxiety disorder.^53^ The KICA-Dep is a screening tool for depression among older Aboriginal Australians.^54^ The GSGL tool is the first culturally sensitive quality of life instrument for older Aboriginal Australians, measuring 12 separate factors that contribute to Elders’ quality of life.^34^

#### Physical fitness assessments

Functional fitness will be evaluated using a series of tests. Grip strength^55^ will be measured for both hands using a JAMAR Smart Hand Dynamometer. The Step Test assesses dynamic balance, where the participant repetitively steps one foot on and off a 7.5 cm high step within 15 s, without relying on hand support.^56^ The Sit-to-Stand Test evaluates functional lower limb strength by timing the participant’s ability to stand up and sit down five times as fast as possible from a standard chair.^57^ Agility and leg strength will be assessed through the Timed Up and Go Test, which measures the time it takes for the participant to stand from a chair, walk 3 m and return to a seated position at a comfortable speed.^58^ A 5-metre walk test will measure walking at a comfortable speed which is reliable for assessing function status in a wide range of populations.^59^ To gauge cardiovascular fitness, the 2 minute walk test involves the participant walking as far as possible along a standardised path in 2 min.^60^ Heart rate will be recorded at 1 and 2 min, with peak heart rate determined using the Polar Heart Rate M200 Monitor connected to a Polar H10 heart rate sensor worn by the participant around the lower chest. An RPE^61^ measure will record participants’ perceived exertion after the completion of the walk.

#### Process evaluation

The DAMPAA process evaluation will be guided by the ToC framework (figure 2). ToC is a framework to guide programme design and evaluation to better understand how and why a programme works within different contexts (eg, cultural, geographic).^62^ A ToC describes the causal pathways through which a programme is hypothesised to create change and is usually developed collaboratively with key stakeholders.^63^ As described elsewhere,^38^ the ToC development approach was used to develop the DAMPAA ToC framework. The ToC framework has scope for change and potential to develop during programme delivery. As a result, our assumptions, indicators or outcomes are subject to modification and/or removal based on continuous programme evaluation.

Interviews will also be completed with DAMPAA group participants using the adapted Clients’ Perception of the Quality of Chronic Condition Care—Systems Assessment Tool,^64^ and open-ended yarning questions, at the end of the action and maintenance phases. At the end of the action phase (6 months), a yarning circle will be conducted with participants at each site, to gain further insights on the DAMPAA programme.

ToC in-depth interviews will be conducted with project staff at 6-month intervals with open-ended questions covering the key components of the DAMPAA ToC framework.

### Ethics and dissemination

The study was supported by the Yamatji Aboriginal Health Planning Forum and Derbarl Yerrigan Health Service Aboriginal Corporation research subcommittee and approved by the Western Australian Aboriginal Health Ethics Committee (WAAHEC) (HREC 732) and the UWA Human Research Ethics Committee (RA/4/20/4944). Ethics amendments will be communicated with WAAHEC and implemented pending approval.

Study results will be published in peer-reviewed journals and presented at scientific meetings. We will also develop and disseminate a comprehensive DAMPAA toolkit for health services including: (1) guidelines and resources for the programme, (2) education materials and resources on dementia risk management aimed at Aboriginal people at risk, their families and the community and (3) facilitator’s guide and training outline directed at Aboriginal care coordinators and other health professionals on dementia risk factors and management.

### Translation strategies

Steps towards the smooth implementation of this research project have already begun from the project planning stages with the active involvement of the ACCOs. The involvement of service providers as co-researchers, along with our Elders Governance Group, will facilitate the generalisability and translation of successful components of the study across Aboriginal health services. A memory clinic with a specialist geriatrician (coauthor LF) visiting Derbarl Yerrigan Health Service East Perth was established in partnership with the DAMPAA study as a referral pathway for people screened as having possible dementia on the KICA-Cog, and will be open for other referral sources. This service will fill a much-needed gap for the Aboriginal community, which has lacked a memory clinic within a culturally safe venue (ACCO) accessible to older Aboriginal people in Perth. Overall, the findings of this study will provide vital information to guide successful programme translation in the community. This will help inform future dementia prevention strategies to reduce the incidence of dementia among Aboriginal Australians.

## supplementary material

10.1136/bmjopen-2024-088281 online supplemental file 1
