# Supplementary material for: Community-based dementia risk management and prevention program for Aboriginal Australians (DAMPAA): a randomised controlled trial study protocol
Source: BMJ Open. 2024 Sep 13;14(9):e088281. doi: 10.1136/bmjopen-2024-088281 (PMC11404219; doi:10.1136/bmjopen-2024-088281)
Supplement: online supplemental file 1 [file bmjopen-14-9-s001.pdf]

Centre for Aboriginal Medical & Dental Health
The University of Western Australia (M303)
35 Stirling Highway
Crawley, WA 6009
Tel: 6488 4803
Email: dampaa@uwa.edu.au

PARTICIPANT INFORMATION FORM

Project Title**: Dementia Prevention and Risk Management Program for Aboriginal Australians -* ***DAMPAA

***Chief Investigators**: Dr. Kate Smith, Professor Leon Flicker, Professor Dawn Bessarab, Assoc. Professor Dina LoGiudice, Assoc. Professor Kay Cox, Professor Osvaldo Almeida, Dr. Paula Edgill, Professor Keith Hill, Ms. Deborah Woods, Ms Carmel Kickett.*

What is this research about?

We want to see if we can help older Aboriginal people keep memories strong through research run in partnership with local Aboriginal health services and community organisations. This research project will involve regular group activity such as walking and yarning and looking after your general health to see if it helps to keep your heart, brain and memory strong.

Your participation is voluntary

Your participation in this study is voluntary and there will be no cost to you. If you do not want to take part in this study you do not have to. You should feel under no obligation to participate in this study. Choosing not to take part in this study will not affect your current and future medical care in any way. Taking part in this study requires proof of your COVID-19 vaccinations. If you are not yet fully vaccinated but would still like to participate in the study, we ask that you provide your COVID vaccination certificate to the researcher by the date of your first DAMPAA assessment.

Your withdrawal from the study

You are under no obligation to continue with the research study. You may change your mind at any time about participating in the research. People withdraw from studies for various reasons and you don't need to provide a reason.

You can withdraw from the study at any time by completing and signing the '**Participant Withdrawal of Consent Form**'. This form is provided at the end of this document, and is to be completed by you and supplied to the research team if you choose to withdraw at a later date.

If you withdraw from the study, you will be able to choose whether the study will destroy or retain the information it has collected about you. You should only choose **one** of these options. Where both boxes are ticked in error or neither box is ticked, the study will destroy all information it has collected about you.

You should also be aware that where your information has already been analysed and/or the results published, it may not be possible for your information to be withdrawn from the study or destroyed. In such circumstances, your information will continue to form part of the study records and study results. Your privacy will continue to be protected at all times. Withdrawing from the study will not affect your access to Health Services or government benefits, and where relevant, will have no bearing on the medical care you receive.

How will we do this research?

We invite you to be part of this project. We are inviting all Aboriginal and/or Torres Strait Islander people aged over 45 years from Perth and Geraldton to take part in a health and wellbeing program.

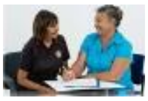

1. With your permission, we will give you a short memory test. Depending on your result we will not ask you to do any more or we will ask you if you would like to keep going to the next part of the study. With your permission we will notify your GP of your results.

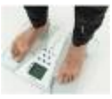

2. We will then ask you about your medical history and we will take some health measures (height, weight, girths, body composition and blood pressure). You will be also asked to agree

to a blood test to check your blood sugar and cholesterol. A researcher will ask you to do some longer memory and wellbeing tests. We will also ask you to do some fitness tests – a two minute walk, a mobility, leg strength, grip strength and balance tests.

If you are happy to continue and your doctor is happy for you to be involved, you will then be put into one of two groups, the *DAMPAA* group or *Usual care* group. The group has to be random – you will not be able to choose which group you are put into. We need to do this to make the study findings as strong as possible.

DAMPAA Group:
0-6 months DAMPAA program

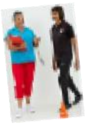

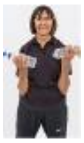

- Physical and social activities two times a week in a group for six months (transport can be organised).
- Physical activity once per week at home or at a community centre.
- A trained exercise instructor and an Aboriginal Health Worker will help you with the program and make sure that it is suitable for your own health and body. They will also talk with you about things that you can do to help improve your heart and brain health.
- Once a week for the first four weeks the Aboriginal Health Worker will check and talk to you about your blood pressure. If blood pressure reading is in recommended range in the first four weeks, it will then be checked once a month. Once a month (or weekly if you are at risk of or have diabetes) we will check your blood sugar levels.

6-12 months DAMPAA program

- Keep going with the physical and social activity program on your own, with regular phone calls from the Aboriginal Health Worker, and you will be retested at 12 months to complete your participation in the study.

Usual care Group:

- We will not ask you to take part in the DAMPAA program, but at 6 and 12 months you will get the same tests as the DAMPAA group.
- We will talk with you about things that you can do to improve your heart and brain health.

All the questionnaires and tests will be repeated at 6 and 12 months to see if anything has changed. During the research project and when it is finished, we will come and yarn with you about what we have found. The findings from the project will be published. No names will be used.

Both groups:

As part of this project we would also like to understand the number of times you go to the doctor or to hospital or use medications or outpatient services, and how much this costs. This information is already collected in your health records by the government through the Medicare Benefits Schedule (MBS) and the Pharmaceutical Benefits Scheme (PBS) and by the WA Department of Health. With your permission, we would like to access this health information to work out if the *DAMPAA* program is cost effective.

We will ask for your permission for the WA Department of Health to provide information about your stays in hospital, visits to a health professionals, your time in the Emergency Department or ambulance services for the 12 month follow up period to Dr Kate Smith for the purposes of the DAMPAA Project. If you agree to this, we will send all information securely to make sure it is safe. We will then combine this information with other information we collect through talking to you.

You will be asked to fill out a consent form authorising the study access to your complete Medicare and Pharmaceutical Benefits Scheme (PBS) data as outlined on the back of the consent form. Medicare collects information on your doctor visits and the associated costs, while the PBS collects information on prescription medications you have filled at pharmacies. The consent form is sent

securely to Services Australia who holds this information confidentially.

To evaluate the project at 6 months and when you leave the program we will ask you some questions about your thoughts on the quality of the program, and how it may be improved. We will also ask you about your thoughts on the DAMPAA health and memory assessments used. We will either ask you these questions face to face, or we may contact you on the telephone. With your permission we will audio record your interviews, so that we can record the information that you tell us accurately.

What are the risks?

All of the questionnaire tests used in this study are standard tests used by GPs or in research. The study staff will help to make sure that you are comfortable with the tests. There may be a little bit of bruising or swelling from the needle used in the 6 monthly blood tests but this should get better within a few days. There is always some minimal risk associated with participation in a physical fitness assessment and physical activity, such as fatigue, dizziness, heart problems or experiencing a fall. **This risk will be minimised by having weekly monitoring of health measures such as blood pressure and glucose levels, supervision of your exercise intensity and by introducing activities gradually and in a way that is appropriate for you.**

What are the benefits?

Being in the study might help your doctors and health workers to look after you better. If you agree the doctors will be given the results of your tests to help with any treatments you might need, or already be having. What we learn will also help other older Aboriginal Australians – if the program is helpful we will be making a training package to give to Aboriginal health services and talk to government about funding to continue this program.

To acknowledge your time commitment you will be given a $25 gift card at screening and at the end of each 6 month assessment period.

What do we do with your information?

We use number codes instead of names to store most of your information, including information from Services Australia and WA Department of Health, so that only the people working directly with you will know your name and contact details. Researchers who look at your results later will only see the number codes, not your name. We will keep research data for five years after the study is finished but this data will be stored in locked filling cabinets and in computers with passwords, and whenever possible it will use the code not your name. At the end of five years, all your personal information related to this research project will be destroyed. Anything we publish will not use names, no-one will be able to be identified. If you decide not to continue with the study all of your information will be destroyed. If you have any questions, or do not understand, ask one of the project staff.

The Medicare and Pharmaceutical Benefits Scheme data that we use for this study will not be used for any future research outside the DAMPAA Project.

Contact persons

If you have any queries about the study, or would like to participate or withdraw from the study, please contact the study coordinator by phone on 6488 4803 or by email dampaa@uwa.edu.au.

Concerns or complaints

If you have any concerns about any side effects of the exercise or tests, contact the DAMPAA study coordinator Alex Lalovic by phone on 6488 4803.

This research project has been reviewed by the Western Australian Aboriginal Health Ethics Committee (WAAHEC.) If you have any complaints about how the project is being run, you can contact the study coordinator or you can contact WAAHEC on 9227 1631, or by email at ethics@ahcwa.org.au.

Centre for Aboriginal Medical and Dental Health
The University of Western Australia (M303)
35 Stirling Highway
Crawley, WA 6009
Tel: 6488 4803
Email: dampaa@uwa.edu.au

PARTICIPANT CONSENT FORM

DAMPAA PROJECT – DEMENTIA PREVENTION AND RISK MANAGEMENT PROGRAM FOR ABORIGINAL AUSTRALIANS

I agree that:

1. The research has been explained to me and I have been able to ask questions about it.
2. All the information I give is strictly confidential and will be used for research purposes for the DAMPAA project only.
3. I will participate in the study, I know that I can stop being in the study at any time, and I don't have to give a reason.
4. The study team can give test results to my doctor or clinic, and referrals made to other health or community services where needed.

   ☐ Yes ☐ No

5. Research staff can access information about my use of health services, including admission to hospital, outpatient services, visits to my GP/specialists, and use of medications.

   ☐ Yes ☐ No

6. Researchers can take blood samples to check my cholesterol and blood sugar only.

   ☐ Yes ☐ No

7. The WA Department of Health can provide information about my stays in hospital, visits to a health professionals, my time in the Emergency Department or ambulance services for the 12 month follow up period to Dr Kate Smith for the purposes of the DAMPAA Project.

   ☐ Yes ☐ No

8. My survey information collected by the DAMPAA Project can be linked to my health related records held by WA Department of Health.

   ☐ Yes ☐ No

9. Researchers can audio record interviews with me for feedback on the DAMPAA study.

   ☐ Yes ☐ No

10. Data collected for the study can be published if information that can identify me is not used
11. I understand that to take part in the DAMPAA project, a copy of my COVID-19 vaccination certificate is required by the date of my baseline assessment.

Name of participant: ______________________________

Medical Centre or GP: ______________________________

Signed:

______________	______	______________	______
Participant	Date	Researcher	Date

This research project has been reviewed by the Western Australian Aboriginal Health Ethics Committee (WAAHEC.) If you have any complaints about how the study is being run, you can contact the trial coordinator 6488 4803 or you can contact WAAHEC at 450 Beaufort St, Highgate 6000, by phone on 9227 1631, or by email at ethics@ahcwa.org.

PARTICIPANT WITHDRAWAL OF CONSENT FORM

Dementia Prevention and Risk Management Program for Aboriginal Australians - DAMPAA

I wish to WITHDRAW my participation in the study effective from the date below. I request that the study handles the information they have collected about me in the following way (choose one option):

☐ DESTROY all information collected about me to date so it can no longer be used for research

☐ RETAIN all information collected about me so it can continue to be used for research

I understand that:

1. no further information about me will be collected for the study from the withdrawal date;
2. information about me that has already been analysed and/or included in a publication by the study, may not be able to be destroyed; and
3. choosing to withdraw from the study will not affect my access to Health Services or Government benefits.

…………………………………………… ……………………………………

Signature **Date**

………………………………………………………..……….

Please print full name

This form should be forwarded by email to: email dampaa@uwa.edu.au. Alternatively, forms can be posted to: Alex Lalovic, University of Western Australia (M303), 35 Stirling Highway, 6009 Perth, Australia
