# Supplementary material for: Community-based dementia risk management and prevention program for Aboriginal Australians (DAMPAA): a randomised controlled trial study protocol
Source: BMJ Open. 2024 Sep 13;14(9):e088281. doi: 10.1136/bmjopen-2024-088281 (PMC11404219; doi:10.1136/bmjopen-2024-088281)
Supplement: Supplementary file 2 [file bmjopen-2024-088281.reviewer_comments.pdf]

PEER REVIEW HISTORY

BMJ Open publishes all reviews undertaken for accepted manuscripts. Reviewers are asked to complete a checklist review form and are provided with free text boxes to elaborate on their assessment. These free text comments are reproduced below.

ARTICLE DETAILS

**TITLE (PROVISIONAL)**	Community-based dementia risk management and prevention program for Aboriginal Australians (DAMPAA): a randomised controlled trial study protocol
**AUTHORS**	Lalovic, Alex; Dowden, Glennette; Markey, Lesley; Bynder, Michael; Yappo, Lynette; Cox, Kay L.; Mateo-Arriero, Irene; Flicker, Leon; Bessarab, Dawn; Thompson, Sandra; Kickett, Carmel; Woods, Deborah; Pestell, Carmela; Edgill, Paula; Hill, Keith; Etherton-Beer, Christopher; Lo Giudice, Dina; Almeida, Osvaldo; Lin, Ivan; Milte, Rachel; Ratcliffe, Julie; Hyde, Zoë; Smith, Kate

VERSION 1 - REVIEW

**REVIEWER NAME**	Osaki, Tohmi
**REVIEWER AFFILIATION**	Kobe Gakuin University Faculty of Rehabilitation, Department of Occupational Therapy
**REVIEWER CONFLICT OF INTEREST**	None
**DATE REVIEW RETURNED**	16-May-2024

**GENERAL COMMENTS**	I sincerely thank you for the opportunity to review this excellent manuscript. This study is well-designed and very interesting, providing a co-designed intervention that is culturally relevant to Aboriginal and Torres Strait Islander peoples (Aboriginal Australians). I look forward to seeing the results of this study.<br>I make a few comments, so please check the contents and consider revising your manuscript if necessary.<br><br>ABSTRACT<br>p2 L29- Methods and analysis<br>The section needs to be completed and should be more detailed. You should at least indicate the Aim, participants, duration of intervention, and outcomes.<br><br>INTRODUCTION<br>p3 L26-<br>The very high incidence of dementia in Aboriginal Australians is mentioned, and several modifiable risk factors are described in the paragraphs that follow. However, it is not sufficient for readers unfamiliar with Aboriginal Australians to understand which of their characteristics are associated with such a high incidence. It would be better for the reader if background information, such as the prevalence of cardiovascular and metabolic diseases and the ageing rate of the population, for example, were presented in numerical values.<br><br>p3 L33<br>Since there is currently a disease-modifying drug for AD, Lecanemab, the sentence "While there is currently no cure for

	dementia..." is a bit inaccurate. Of course, the benefits of that drug are minor, and side effects are not infrequent so that non-pharmacologic therapy will remain the optimal approach for people with dementia and MCI.<br><br>METHODS<br>p5 L57-, p10 L9-, Table 3, and Fig 1<br>The description of the eligibility criteria needs to be clarified. For example, on page 5, there is no mention of KICA-cog, KICA-function or BMI among the inclusion and exclusion criteria. However, on page 10, it is stated that these are the eligibility criteria. The same page also states that researchers will decide to proceed to Visit 2 after all the neuropsychological tests of Visit 1 have been performed; it may indicate the "Exclusion & referral: possible dementia or depression" flow in Fig. 1, but there is no mention of depression as an exclusion criterion.<br>I would suggest reorganising it into a flow of 'eligibility assessment', 'screening', 'enrolment' and 'baseline assessment'.<br><br>p8 L53- Sample size<br>You have predicted the changes in the KICA-cog, but you should provide evidence. Also, the sample size calculation requires a pooled standard deviation for the effect size calculation.<br>I make one more comment in this section: based on the FINGER trial, many other dementia prevention RCTs, and my own experience, I expect cognitive function tests every six months to increase scores in both groups. What are your thoughts on this point?<br><br>p9 L52 ...over time between the two groups.<br>The primary endpoint should specify the change at 6 months or 12 months. Please clarify at which point in time group differences represent efficacy, which is your primary research objective.<br><br>p9 L53<br>Should recruitment sites be included in the random effect (or covariate)? Also, what are your thoughts on considering age groups in the statistics?<br>This study is an excellent initiative in that it intervenes with older adults and those in middle age. However, there are subtly different risk factors for dementia in middle age and older age (For example, whether blood pressure or cholesterol levels in older people linearly increase risk is debatable), and the responsiveness to interventions is different. Careful allocation and statistical analysis are needed.<br><br>p9 L57<br>I'm sorry, but I did not understand the intention of this sentence: 'We will handle missing data by performing complete case analyses.' Is this inconsistent with ITT analysis and mixed-model analysis?<br><br><br>I want to comment on a few minor points below.<br><br>p2 L5: Please check that the 'Australian New Zealand Clinical Trials Registry' abbreviation is correct as 'ACTRN' (ANZCTR?).<br><br>Multiple places in the text: Please check if you can describe the whole group of participants (i.e. including middle-aged people) as 'older'.

	p5 L13: There is no description of the evaluation of 'cost-effectiveness'; please delete this phrase or add an explanation in the text.<br><br>Table 1: Is the '6-minute walk test' a mistake for the '2-minute walk test'?<br><br>References: Please check that it conforms to the journal's prescribed style.<br><br>**I sincerely wish you success in this research!**

**REVIEWER NAME**	Gatz, Margaret
**REVIEWER AFFILIATION**	University of Southern California, Department of Psychology
**REVIEWER CONFLICT OF INTEREST**	no competing interests
**DATE REVIEW RETURNED**	21-May-2024

**GENERAL COMMENTS**	The authors have done an impressive job of designing a dementia risk management program that is premised on Aboriginal culture and that builds extensive partnerships with Aboriginal communities and leadership. Given how far the study has already advanced, there is no real chance to make changes to the methodology of the intervention and data collection. However, there are places where the manuscript could be clearer about rationale or details.<br>A first question is about the scope and intensity of the intervention. The two main components are walking and yarning. There are two sessions per week, each up to 90 minutes. Does each 90-minute session comprise walking plus yarning? Does that mean a total of [up to] 3 hours of intervention per week? Medication management might be considered a third main component; therefore, “dose” of medication management would be a useful variable as well, e.g., whether a cholesterol medication was changed.<br>Health education is delivered in a yarning style, presumably as part of that same 90 minutes. Other than presenting information, what theory of change principles will be applied to improving nutrition, falls prevention skills, community services access, etc.? Blood pressure is a secondary outcome; will it be included in the health education program?<br>A second question is about the usual care group. At the conclusion of the RCT, will they have an opportunity to experience DAMPAA? Is there a plan to assess whether they have engaged in any other dementia risk management program during the year between the first assessment and the final assessment, or even whether they may have changed their habits in response to the informational pamphlet that constitutes usual care? I understand that you would not want to monitor weekly because that would constitute an intervention.<br>A further question is with respect to recruitment. Do the ACCOs who are referring people to the program understand that only half of those referred will receive the intervention? When the people sign up, prior to randomization, what are they told about their likelihood of in fact receiving the intervention for which they volunteered?<br>It appears from the description of randomisation that possibly each site will have both DAMPAA and usual care. If that is the case, what steps are being taken to prevent experimental contamination? Relatedly, if an ACCO refers a couple for the program, could husband and wife be randomised to different conditions? If two

	friends were interested in the program, could they be randomised to different conditions? Then, what precautions are taken when the participant is doing the home sessions to assure the DAMPAA participant is not group walking with people from usual care?<br>Also, some questions about the research design and analyses. The inclusion of an action period and a maintenance period provides some test of how much the participants' new habits carry beyond the end of the program. However, during the maintenance period, staff will continue to contact participants and monitor their amount of participation. Is there a plan for a further follow-up in another six months to see how much has carried over?<br>Randomisation is described as being at the individual level. Later it says that randomization is stratified by centre, and that there are three centres. Do the 3 strata or 3 blocks correspond to the 3 centres? The program is also described as multi-site and three sites are listed. Is site the same as centre? There seems to be nothing in data analysis about including centre or site as an effect, which would seem necessary.<br>Analyses are intent-to-treat. It also seems that attrition needs to be studied as part of the overall analytic plan, especially if there is differential attrition by condition. If someone does drop out of DAMPAA, will there be an effort nonetheless to invite to the assessments?<br>The sample sizes are calculated based on a very specific prediction that if no intervention is made, KICA-Cog scores will decrease by a mean of 2 points over 12 months. What is the basis for that prediction? It is also predicted that the intervention will reduce the score decrease to 1 point. It makes sense to posit a reduction in cognitive decline as an outcome rather than an increase in cognitive scores. But, again, what is the basis for predicting one point? There is similar specificity with respect to daily function, again without spelling out the basis for the prediction.

VERSION 1 – AUTHOR RESPONSE

Reviewer 1

Dear Authors

I sincerely thank you for the opportunity to review this excellent manuscript. This study is well-designed and very interesting, providing a co-designed intervention that is culturally relevant to Aboriginal and Torres Strait Islander peoples (Aboriginal Australians). I look forward to seeing the results of this study.

I make a few comments, so please check the contents and consider revising your manuscript if necessary.

ABSTRACT

p2 L29- Methods and analysis

The section needs to be completed and should be more detailed. You should at least indicate the Aim, participants, duration of intervention, and outcomes.

Response: The requested changes have been made.

INTRODUCTION

p3 L26-

The very high incidence of dementia in Aboriginal Australians is mentioned, and several modifiable risk factors are described in the paragraphs that follow. However, it is not sufficient for readers unfamiliar with Aboriginal Australians to understand which of their characteristics are associated with such a high incidence. It would be better for the reader if background information, such as the prevalence of cardiovascular and metabolic diseases and the ageing rate of the population, for example, were presented in numerical values.

Response: We have added the following to the manuscript:

In 2018-19, 31% of Aboriginal and Torres Strait Islander adults had high blood pressure and 13% had diabetes or hyperglycaemia, while 71% of those aged ≥15 years were overweight or obese and 37% were daily smokers. Only 12% of adults living in non-remote areas met physical activity guidelines (16).

p3 L33

Since there is currently a disease-modifying drug for AD, Lecanemab, the sentence "While there is currently no cure for dementia..." is a bit inaccurate. Of course, the benefits of that drug are minor, and side effects are not infrequent so that non-pharmacologic therapy will remain the optimal approach for people with dementia and MCI.

Response: We prefer to leave this sentence unchanged. Although the disease-modifying drug lecanemab has been approved for use in the United States of America (with a black box warning of serious adverse events), it has not received approval for use in Australia outside of clinical trials. Further, although lecanemab may slow the progression of cognitive decline in people with early Alzheimer’s disease, it is not a cure.

METHODS

p5 L57-, p10 L9-, Table 3, and Fig 1

The description of the eligibility criteria needs to be clarified. For example, on page 5, there is no mention of KICA-cog, KICA-function or BMI among the inclusion and exclusion criteria. However, on page 10, it is stated that these are the eligibility criteria. The same page also states that researchers will decide to proceed to Visit 2 after all the neuropsychological tests of Visit 1 have been performed; it may indicate the "Exclusion & referral: possible dementia or depression" flow in Fig. 1, but there is no mention of depression as an exclusion criterion.

I would suggest reorganising it into a flow of 'eligibility assessment', 'screening', 'enrolment' and 'baseline assessment'.

Response: We have clarified the inclusion and exclusion criteria, which now read:

Inclusion criteria include: (1) Aboriginal person; (2) aged between 45 and 90 years; (3) community-dwelling; and (4) normal cognition (KICA-Cog score 38-39) or cognitive impairment without dementia (KICA-Cog score 31-37 without functional decline due to dementia). Exclusion criteria include: (1) dementia diagnosis; (2) presence of medical condition that restricts walking without assistance; (3) unstable or life-threatening medical condition; and (4) medical condition that contraindicates moderate physical activity, including morbid obesity (BMI ≥40 kg/m^2).

p8 L53- Sample size

You have predicted the changes in the KICA-cog, but you should provide evidence. Also, the sample size calculation requires a pooled standard deviation for the effect size calculation.

I make one more comment in this section: based on the FINGER trial, many other dementia prevention RCTs, and my own experience, I expect cognitive function tests every six months to increase scores in both groups. What are your thoughts on this point?

Response: As previously noted in a response to the editor’s comments, we have now stated that the expected changes are based on unpublished data from one of our cohort studies of older Aboriginal people. We have also rephrased the description of the sample size calculation to reflect that a common standard deviation is assumed. The text now reads:

Based on unpublished analyses of a cohort study of ageing well (7), we expect KICA-Cog scores to decrease by a mean of 2 points in the usual care group, and by 1 point in the DAMPAA program group, with a common standard deviation of 3 points.

We did not explicitly consider practice effects in the sample size calculation, but it is likely that participants’ scores might increase somewhat over time as a result of practice effects. However, regardless of practice effects, we would still expect the intervention group to decline less than the control group, and this study was powered to detect a minimum difference between each group’s change of 1-unit.

p9 L52 ...over time between the two groups.

The primary endpoint should specify the change at 6 months or 12 months. Please clarify at which point in time group differences represent efficacy, which is your primary research objective.

Response: We have amended this sentence to read:

We will use a generalised linear mixed model to compare changes in the KICA-Cog (cognition, primary outcome measure) over time between the two groups, with a significantly greater decrease in KICA-Cog scores expected in the usual care group at 12 months.

p9 L53

Should recruitment sites be included in the random effect (or covariate)? Also, what are your thoughts on considering age groups in the statistics?

This study is an excellent initiative in that it intervenes with older adults and those in middle age. However, there are subtly different risk factors for dementia in middle age and older age (For example, whether blood pressure or cholesterol levels in older people linearly increase risk is debatable), and the responsiveness to interventions is different. Careful allocation and statistical analysis are needed.

Response: We have added the following to the statistical analysis section of the manuscript:

Participant and site will be treated as random effects.

We agree that it would be useful to explore the effect of age, although we are not certain that we will have sufficient statistical power to undertake such analyses and hence do not pre-specify this aspect of the analysis in our protocol.

p9 L57

I'm sorry, but I did not understand the intention of this sentence: 'We will handle missing data by performing complete case analyses.' Is this inconsistent with ITT analysis and mixed-model analysis?

Response: This is not inconsistent with either intention-to-treat analysis or mixed-model analysis. It will not be possible to include participants in the analysis if they lack data for key variables such as their KICA-Cog score. To clarify what we mean by intention-to-treat analysis, we have changed the manuscript to read as follows:

The study will employ an intention-to-treat analysis including all randomised participants. This means that participants will be analysed as per their randomisation, rather than the treatment they ultimately receive.

I want to comment on a few minor points below.

p2 L5: Please check that the 'Australian New Zealand Clinical Trials Registry' abbreviation is correct as 'ACTRN' (ANZCTR?).

Response: Thank you for identifying this incorrect abbreviation. We have corrected it.

Multiple places in the text: Please check if you can describe the whole group of participants (i.e. including middle-aged people) as 'older'.

Response: Aboriginal and Torres Strait Islander people experience age-related conditions at an earlier age than the wider Australian population and can access government-funded aged care programs at 50 years of age, with Aboriginal and Torres Strait Islander people who are homeless able to access aged care from 45 years.

p5 L13: There is no description of the evaluation of 'cost-effectiveness'; please delete this phrase or add an explanation in the text.

Response: We have removed the sentence about cost-effectiveness.

Table 1: Is the '6-minute walk test' a mistake for the '2-minute walk test'?

Response: Thank you for identifying this mistake, which we have now corrected.

References: Please check that it conforms to the journal's prescribed style.

Response: The references have been updated to match the journal’s style.

Reviewer 2

The authors have done an impressive job of designing a dementia risk management program that is premised on Aboriginal culture and that builds extensive partnerships with Aboriginal communities and leadership. Given how far the study has already advanced, there is no real chance to make changes to the methodology of the intervention and data collection. However, there are places where the manuscript could be clearer about rationale or details.

A first question is about the scope and intensity of the intervention. The two main components are

walking and yarning. There are two sessions per week, each up to 90 minutes. Does each 90-minute session comprise walking plus yarning? Does that mean a total of [up to] 3 hours of intervention per week? Medication management might be considered a third main component; therefore, "dose" of medication management would be a useful variable as well, e.g., whether a cholesterol medication was changed.

Response: The description of the DAMPAA program has been amended to read:

During the action period, participants will attend two group walking and yarning sessions per week at an ACCO and complete a third exercise-only session at home. Each session will be up to 90 minutes in duration. Participants will therefore be expected to engage in up to 270 minutes of DAMPAA program activity per week.

Medication management is indeed an outcome of interest and is included in Table 2. We have modified the manuscript to provide more detail about this outcome as follows:

Medication reviews will be conducted by a pharmacist during the action period in a face-to-face meeting with each participant at the relevant study site. Each participant's general practitioner (GP) will be sent a letter detailing the outcome of the review, which may include recommendations for medication changes. The study coordinator will contact GPs to determine if recommended changes were made.

Health education is delivered in a yarning style, presumably as part of that same 90 minutes. Other than presenting information, what theory of change principles will be applied to improving nutrition, falls prevention skills, community services access, etc.? Blood pressure is a secondary outcome; will it be included in the health education program?

Response: Health education topics are to be delivered once every four weeks during the 90-minute walking and yarning session. Falls prevention and balance exercises are part of the walking and yarning program, aimed at improving balance skills and reducing likelihood of falls. Blood pressure management will not be a formal part of the health education program, but participants' blood pressure will be monitored throughout the trial.

A theory of change (ToC) approach was used to design the trial in a co-design process with stakeholders, including Aboriginal Elders and health services. It will also be used to guide the process evaluation. However, a ToC approach will not be used to deliver the DAMPAA program to participants. The reviewer may possibly be confusing the ToC approach with theories and models of behaviour change, such as the Stages of Change Model (Transtheoretical Model) commonly used in health promotion.

A second question is about the usual care group. At the conclusion of the RCT, will they have an

opportunity to experience DAMPAA? Is there a plan to assess whether they have engaged in any other dementia risk management program during the year between the first assessment and the final assessment, or even whether they may have changed their habits in response to the informational pamphlet that constitutes usual care? I understand that you would not want to monitor weekly because that would constitute an intervention.

Response: We do not plan to assess whether participants have engaged in any other dementia management program during the course of the DAMPAA trial or enquire about other behaviour changes which may have taken place. All participants will be offered the chance to experience the DAMPAA program.

We have modified the manuscript to read:

On conclusion of the trial, we plan to offer participants in the usual care group a chance to experience the DAMPAA program, which will continue to be delivered by ACCOs.

A further question is with respect to recruitment. Do the ACCOs who are referring people to the

program understand that only half of those referred will receive the intervention? When the people sign up, prior to randomization, what are they told about their likelihood of in fact receiving the intervention for which they volunteered?

Response: The DAMPAA study was co-developed with the participating ACCOs, who understood that it was a randomised controlled trial, and that only half of participants would receive the intervention.

Potential participants were informed that they would be randomised into either the DAMPAA program or the usual care group. They were also informed that it was not possible to choose which group they would be randomised to. The Participant Information Sheet (which is now provided as Supplementary Material) clearly explained what being in either of these groups would involve.

It appears from the description of randomisation that possibly each site will have both DAMPAA and usual care. If that is the case, what steps are being taken to prevent experimental contamination?

Response: Staff delivering the DAMPAA program at the participating ACCOs were not blinded to the intervention. This meant that they could ensure that the group walking and yarning sessions were only delivered to people randomised to the intervention group.

Relatedly, if an ACCO refers a couple for the program, could husband and wife be randomised to

different conditions? If two friends were interested in the program, could they be randomised to

different conditions? Then, what precautions are taken when the participant is doing the home sessions to assure the DAMPAA participant is not group walking with people from usual care?

Response: Given the extended family structure of Aboriginal people and importance of community, it may not be possible to prevent experimental contamination. This limitation is now acknowledged in the manuscript.

Also, some questions about the research design and analyses. The inclusion of an action period and a maintenance period provides some test of how much the participants' new habits carry beyond the end of the program. However, during the maintenance period, staff will continue to contact participants and monitor their amount of participation. Is there a plan for a further follow-up in another six months to see how much has carried over?

Response: No further follow-up is planned beyond 12 months.

Randomisation is described as being at the individual level. Later it says that randomization is stratified by centre, and that there are three centres. Do the 3 strata or 3 blocks correspond to the 3 centres? The program is also described as multi-site and three sites are listed. Is site the same as centre? There seems to be nothing in data analysis about including centre or site as an effect, which would seem necessary.

Response: We have changed the wording of the randomisation section so that a "centre" is now described as a "site", ensuring consistency with the rest of the manuscript. As per a previous response to Reviewer 1, the statistical analysis section now notes that a random effect will be included for site.

Analyses are intent-to-treat. It also seems that attrition needs to be studied as part of the overall analytic plan, especially if there is differential attrition by condition. If someone does drop out of DAMPAA, will there be an effort nonetheless to invite to the assessments?

Response: Where a participant is lost to follow-up (but has not indicated that they wish to withdraw from the study) we will make every effort to locate that participant so that they can be included in the trial analyses. As we now note in the data management and analysis section of the manuscript, results of the study will be reported in accordance with the Consolidated Standards of Reporting Trials (CONSORT) statement. This will include details of attrition.

The sample sizes are calculated based on a very specific prediction that if no intervention is made, KICACog scores will decrease by a mean of 2 points over 12 months. What is the basis for that prediction? It is also predicted that the intervention will reduce the score decrease to 1 point. It makes sense to posit a reduction in cognitive decline as an outcome rather than an increase in cognitive scores. But, again, what is the basis for predicting one point? There is similar specificity with respect to daily function, again without spelling out the basis for the prediction.

Response: As noted in a previous response to Reviewer 1, these predicted changes are based on observations from one of our cohort studies of ageing well in older Aboriginal people.

We hope these amendments have improved the manuscript to your satisfaction and thank you for your further consideration of this work.

With kind regards,

Yours sincerely,

Alex Lalovic (on behalf of all authors)
